# Parkinson's disease related signal change in the nigrosomes 1–5 and the substantia nigra using T2* weighted 7T MRI

**DOI:** 10.1016/j.nicl.2018.05.027

**Published:** 2018-05-24

**Authors:** Stefan Theodor Schwarz, Olivier Mougin, Yue Xing, Anna Blazejewska, Nin Bajaj, Dorothee P. Auer, Penny Gowland

**Affiliations:** a Radiological Sciences, Division of Clinical Neuroscience, School of Medicine, University of Nottingham, Queen's Medical Centre, Nottingham, UK; b Sir Peter Mansfield Imaging Centre, University of Nottingham, Nottingham, UK; c Department of Neurology, Queen's Medical Centre, Nottingham University Hospitals, Nottingham, UK; d Department of Radiology, Cardiff and Vale University Health Board, Cardiff, UK; e Cardiff University Brain Research Imaging Centre (CUBRIC), University of Cardiff, Cardiff, UK; f NIHR Nottingham Biomedical Research Centre, Nottingham, UK

**Keywords:** Nigrosome, MRI, Parkinson's disease, Substantia Nigra, T2*

## Abstract

Improved markers for the progression of Parkinson's disease (PD) are required. Previous work has proven that iron dependent MRI scans can detect the largest Nigrosome (N1) within the substantia nigra (SN) pars compacta and changes in PD. Histopathological studies have shown that N1 is particularly affected in early PD whereas the other nigrosomes (N2–N5) and the surrounding iron-rich SN are affected later. In this study we aimed to determine whether MRI can detect the smaller nigrosomes (N2–N5) and whether graded signal alterations can be detected on T2*-weighted MRI at different disease stages consistent with histopathological changes.

An observational prospective study was performed within the research imaging centre at the University of Nottingham, UK. Altogether 26 individuals with confirmed PD (median Hoehn&Yahr stage = 1, Unified PD Rating Scale [UPDRS] = 12.5) and 15 healthy controls participated. High resolution T2*weighted 7T MRI of the brain was performed and visibility of N1-N5 within the SN was qualitatively rated. Normalised T2*weighted signal intensities in manually segmented N1–N5 regions and iron-rich SN were calculated. We performed group comparisons and correlations with severity based on UPDRS. Qualitative measures were a nigrosome visibility score and a confidence score for identification. Quantitative measures were T2*weighted contrast of N1–5 and iron-rich SN relative to white matter.

We found that visual assessment of the SN for N1–N5 revealed normal range visibility scores in 14 of 15 controls. N1 was identified with the highest confidence and visibility was in abnormal range in all 26 PD patients. The other nigrosomes were less well visible and less confidently identified. There was a larger PD induced signal reduction in all nigrosomes than in the iron-rich SN (median signal difference N1–5 PD compared to controls: 19.4% [IQR = 24%], iron-rich SN 11% [IQR = 24%, *p* = 0.017]). The largest PD induced signal reduction was in N1: 37.2% [IQR = 19%] which inversely correlated with UPDRS in PD (R^2^ = 0.19).

All nigrosomes can be detected using 7T MRI, and PD induced T2*weighted signal reduction was greatest in the nigrosomes (especially N1). The graded T2*weighted signal alterations in the nigrosomes match previously described differential histopathological effects of PD. N1 was identified with the highest confidence and T2*weighted signal in N1 correlated with UPDRS confirming N1 as the most promising SN marker of PD pathology.

## Highlights

• Parkinson's disease induced nigrosome1–5 alterations are investigated using T2* 7T-MRI.
• Disease induced changes are seen in all nigrosomes and the surrounding nigra.
• Nigrosome1 is most confidently and most reliably identified.
• Signal in N1 demonstrates highest correlation with disease severity related change.

## 1 Introduction

The loss of dopaminergic neurons of the substantia nigra pars compacta (SNpc) is a well-known early histological characteristic of Parkinson Disease (PD). Using the immunohistochemical compounds against Calbindin D28K and Tyrosine Hydroxylase the SNpc can be segmented into a Calbindin positive iron-rich SN and five Calbindin negative nigrosomes (N1 to N5) (Damier et al., 1999a). Nigrosomes contain clusters of dopaminergic cells and are sequentially affected throughout the progression of PD (Damier et al., 1999b). Nigrosomes have lower iron content than the surrounding iron-rich SN in healthy subjects and the largest nigrosome N1 has previously been identified as a small, high signal intensity region in the posterior SN on T2* weighted (T2*w) or other iron sensitive MRI sequences at 7Tesla (Blazejewska et al., 2013; Cosottini et al., 2014) and 3 Tesla (Cosottini et al., 2015; Reiter et al., 2015; Schwarz et al., 2014). PD induced cell loss and associated increased iron deposition causes MRI signal reduction in N1 allowing differentiation of early stage PD from non-PD with high accuracy at 7T (Blazejewska et al., 2013; Cosottini et al., 2014, Cosottini et al., 2015) and 3 T (Cosottini et al., 2014, Cosottini et al., 2015; Mahlknecht et al., 2017; Noh et al., 2015; Reiter et al., 2015; Schwarz et al., 2014). The ‘swallow tail sign’ has been proposed as a memorable radiological sign to describe the appearance of the healthy N1 on 3 T axial T2*w MRI, a feature that is lost in PD (Schwarz et al., 2014). Post mortem investigations have confirmed that all five nigrosomes can be demonstrated using ultra-high field 9.4 T MRI (Massey et al., 2016) in both healthy and PD affected brains.

The physiological and functional relevance of the different nigrosomes is unknown, but from histological studies regional differences in the time course of PD induced loss of dopaminergic cells across the individual nigrosomes can be inferred. The cells of N1 are affected early, sequentially followed by cells in N2, N4, N3 and finally N5 in the later disease stages (Damier et al., 1999b). This is also supported by findings from post mortem studies using alternative algorithms for SN segmentation that are independent of nigrosomes. PD induced neuronal loss is greatest (averaging 91%) in the region described as ‘lateral ventral tier’ which topographically overlaps with N1 (Fearnley and Lees, 1991) whereas average cell loss in regions termed the ventral and dorsal tiers was smaller (71% and 56% respectively) which corresponds to the nigral regions where N2–N5 can be found.

Pathological iron accumulation throughout the substantia nigra has long been established in PD (Sofic et al., 1988). Although the cause of the iron increase is poorly understood it has been hypothesised that unbound iron contributes to free hydroxyl radicals, which may cause lipid per-oxidation and contribute to dopaminergic cell death (Dexter et al., 1989). Dopaminergic neurons of the SN also contain neuromelanin pigment, a dark protein polymer, which is implicated in the iron homeostasis (Zecca et al., 2008). PD induced iron changes in the SN result in signal alterations on iron sensitive T2, T2*, T2’ and inversely correlated R2, R2* and R2’ weighted MRI sequences (Gorell et al., 1995; Lotfipour et al., 2012). However, it is unclear if there is preferential progression of iron related signal changes in the nigrosomes or in the adjacent iron-rich SN during the course of the disease. The pathological iron deposition in the individual nigrosomes may follow a similar sequential pattern as the dopaminergic cell loss throughout different disease stages (Damier et al., 1999b).

This study aimed to determine whether all five nigrosomes could be visualized in vivo and to investigate whether there was a specific disease severity dependent pattern of T2*w signal attrition on ultra-high field (7T) high resolution T2*w MRI in the substantia nigra and nigrosomes which matched changes previously reported in histopathological studies. This would be an important step in refining this MRI biomarker to detect very early pathological and progressive PD changes.

## 2 Methods

### 2.1 Standard protocol approval and patient consent

This study and the study protocols were approved by the institutional review board and the local Research Ethics Committee (National Research Ethics Service, Derbyshire Research Ethics Committee). All participants gave informed consent before enrolment into the study and were offered reimbursement of travel cost for the study. Participants did not receive further compensation. The study was conducted from May 2011 to November 2015 and analysis was performed periodically from October 2014 to January 2017.

### 2.2 Participants

Participants were prospectively recruited from local movement disorder clinics of the Nottingham University Hospitals NHS Trust and Royal Derby Hospital NHS Trust in England. Twenty six PD patients (8 females) with confirmed PD (average age of 64.8, median UPDRS: 12.5, median Hoehn&Yahr score: 1, median disease duration: 1.5 years) and 15 controls (10 females, average age: 65.3) were recruited (list of participant details see supplementary data: Appendix A). Controls were recruited from spouses/friends of patients when attending the same service or through advertisements within Nottingham University Hospitals. PD patients were diagnosed clinically upon fulfilment of the UK PD Brain Bank clinical criteria (Hughes et al., 1992) or in case of diagnostic uncertainty (16 subjects) by additional usage of an ioflupane iodine-123 single photon emission tomography (DaTscan™). All patients were seen by a movement disorder specialist consultant (N.B.). The Unified Parkinson's disease Rating Scale (UPDRS) rating was performed by a neurology consultant (N.B.) or clinical research nurse (Charlotte Downes) with >10 and 2 years of clinical PD research respectively. The term UPDRS used in the manuscript always refers to the total UPDRS score (Section I – IV of the UPDRS) according to Fahn et al. (1987). MRI scans from three participants (two PD) had to be excluded due to poor quality relating to susceptibility/movement artefacts. Twenty-five of 26 PD patients and all 15 healthy controls were assessed for cognitive impairment by usage of the Addenbrooke's cognitive examination (ACE) test battery (Bak and Mioshi, 2007). PD patients had a significantly lower ACE score than controls (average ± std., patients: 91.1 ± 6.5; controls: 96.5 ± 3.6; *t*-test, *p* = 0.006). The mini mental test score (MMSE) was borderline not significantly different (patients: 27.8 ± 2.2, controls 29 ± 1.7, *t*-test, *p* = 0.08).

### 2.3 MRI protocol and image analysis

Magnetic resonance imaging was performed on a 7T Philips Achieva scanner using a 32-channel receiver coil. Following previous experience in patient scanning and to minimize motion artefact, a 2D T2*w sequence optimized to image the substantia nigra at 7T was used (Blazejewska et al., 2013). High resolution T2*w MRI was obtained using a 2D Fast-Field Echo sequence with TE/TR = 16/412 ms, nominal flip angle α = 40°, no sense, 2 signal averages, Field of View = 180 × 160 × 16 mm^3^, and 0.35 × 0.35 × 1 mm^3^ resolution in 9.5 min.

**Fig. 1:**
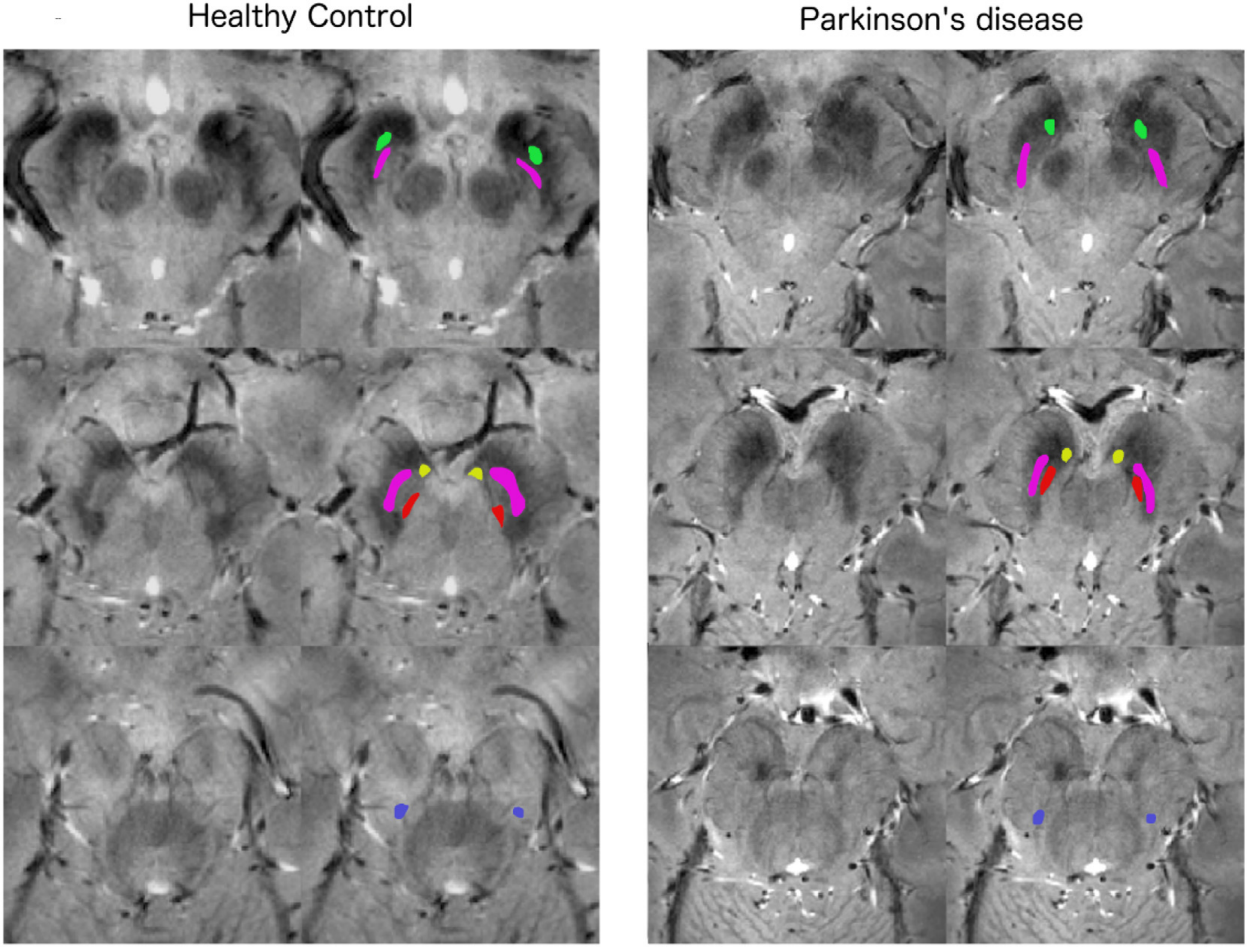
Identification and ROI placement of N1 to N5 in controls and PD. Sample images of the rostral, medial and caudal substantia nigra before and after ROI placement in the five nigrosomes in a healthy control (first two columns, age = 65 years, male) and a patient with PD (column three and four, age = 60 years, male, UPDRS 10, disease duration = 1 year, HY score 1). The ROIs placed in the regions of the different nigrosomes are colour coded with N1 = purple, N2 = yellow, N3 = blue, N4 = red and N5 = green.

A neuroradiologist (STS) blinded to subject information scored the detectability of nigrosomes 1–5 on a 6 point nigrosome visibility scale (VS: 5 = normally bright and present; 4 = slightly more difficult to see than normal/reduced size - but definitely identifiable, 3 = very difficult to see but identifiable, 2 = possibly parts of the outline visible but not definitely identifiable, 1 = not identifiable as not different from surrounding low signal, 0 = darker than surrounding SN). Unilateral or bilateral VS of 0–2 were considered pathological. The rater also drew ROIs outlining the nigrosomes. If it was difficult to definitely identify the nigrosome (VS = 1–2) ROIs were placed in the expected position of the individual nigrosomes (Fig. 1). Confidence in how well the nigrosomes could be identified was also scored on a 3 points confidence scale ([CS], 1 = high confidence, 2 = moderate confidence, 3 = low confidence). The T2*w signal in the nigrosome ROIs was normalised by signal from a local white matter (WM) region, drawn at the same level as the red nucleus. Signals from the right and left sides were averaged. T2*w of the iron-rich SN was derived by averaging signal from a seed-based automatic segmentation with manual refinement (Neuroi: https://www.nottingham.ac.uk/research/groups/clinicalneurology/neuroi.aspx) based on signal intensity of the SN with nigrosome ROIs removed, and was normalised to local WM of the brainstem tegmentum.

All images were registered to a final near-isotropic template of 0.67 × 0.67 × 0.7 mm using FLIRT from FSL, using a sinc-interpolation to minimize blurring and to facilitate the segmentation of the nigrosomes in 3D. For inter- and intra- rater reliability assessment of the nigrosome- segmentation ten random scans from the data set were blindly scored by two raters (STS–9 years of experience and YX–4 years of experience in PD imaging research) and twice by one of the raters (STS).

### 2.4 Statistics

All results such as confidence scale, visibility and T2*w normalised signal are presented as median and interquartile range. Relative signal changes between healthy controls and PD were calculated in relation to the median value observed in the healthy control group (Median-percent-change) to account for outliers and skewed distributions (Geraci et al., 2013). In some parts of the analysis the UPDRS in controls was assumed to be 0 without assessment. Patients were arbitrarily grouped into two severity groups according to the UPDRS (≤10 [*n* = 10] “very early PD” and >10 [*n* = 16] “early to moderate PD) or for an “overview colour-chart” of SN signal (Fig. 4) into three severity groups (UPDRS ≤ 10 [n = 10], 11–30 [*n* = 9], >30 [*n* = 7]).

All statistics were produced using SPSS (V23) or the Curve Fitting Toolbox™ in Matlab (The MathWorks Inc., Natick, MA). *t*-Tests were used for normally distributed data, Mann-Whitney *U* test for non-normally distributed data. The relationship between UPDRS and the normalised T2*w data was assessed using linear regression or a simple exponential regression analysis using the Pearson correlation coefficient. The tests were corrected for multiple comparison describing Bonferroni corrected *p*-values when necessary. The intraclass correlation coefficient (ICC) was used to measure the degree of consistency among ratings made by one investigator twice and two different investigators on visibility scores of the nigrosomes.

## 3 Results

### 3.1 Nigrosome visibility

All five nigrosomes could be visualized in both SNpc in 14 of 15 control subjects (VS of 5–3). The visibility of N1 was high in controls (VS of 5–3, 14/15, both SNpc) and low in all 26 PD patients (VS of 2–0). The visibility of N2–N5 was poorer than the visibility of N1 in controls and there was a smaller difference between the visibility scores of N2-N5 in controls and patients when compared to N1 (Table 1). This translates into a sensitivity, specificity and accuracy of 100%, 93% and 98% respectively for using the visibility of N1 on high resolution T2*w 7T MRI to differentiate PD from controls, using defining VS ≤ 2 as abnormal (see methods). VS for N2–N5 were less reliable in differentiating PD and controls with a lower inter- and intra-rater reproducibility (Table 1).

**Table 1:** Visibility of nigrosomes 1–5, confidence of NS identification and reproducibly of NS scores in Parkinson's and controls.

Nigrosome	Visibility			Confidence			ICC	
	HC (IQR)	PD (IQR)	Mann W U	HC (IQR)	PD (IQR)	Mann W U	Inter	Intra
N1	5 (0)	1 (0.75)	*p* < 0.001	1 (0)	2 (0)	*p* < 0.001	0.84	0.98
N2	4 (2)	3(3)	*p* < 0.001	2 (2)	3 (1)	*p* = 0.001	0.4	0.57
N3	4 (1.25)	2 (2)	*p* < 0.001	3 (1)	3 (0)	*p* = 0.007	0.53	0.59
N4	4 (1)	4 (1)	*p* = 0.05	1.5 (1)	2 (1)	*p* = 0.007	0.38	0.81
N5	4 (1)	3.5 (1)	*p* = 0.04	2 (0.25)	2 (1)	*p* = 0.044	0.7	0.52

The visibility and confidence score is shown as median in controls (HC) or patients (PD) of each SNpc separate for each nigrosome, together with the Interquartile range (IQR) in brackets. The reproducibility of nigrosome visual scoring was tested using Intra-class correlation measures for the visibility scores (ICC).

### 3.2 Nigrosome identification confidence

The position of N1 was identified with greatest confidence in controls (Median CS = 1, Table 1, Mann-Whitney U vs N2–N5 controls all *P* < 0.001). The confidence was lower for identifying the position of N1 in PD and of the other nigrosomes (Table 1).

### 3.3 Nigrosome identification reliability

The reproducibility of NS visibility scores was assessed in a subset of ten participants (*n* = 20 SN, N1–5) demonstrating a good and excellent inter- and intra-rater reproducibility of the N1 scores respectively. The inter- and intra- rater reproducibility of the N2–N5 scores where lower ranging from fair to good.

### 3.4 Nigrosome and SN T2*w signal in PD compared to controls

**Fig. 2:**
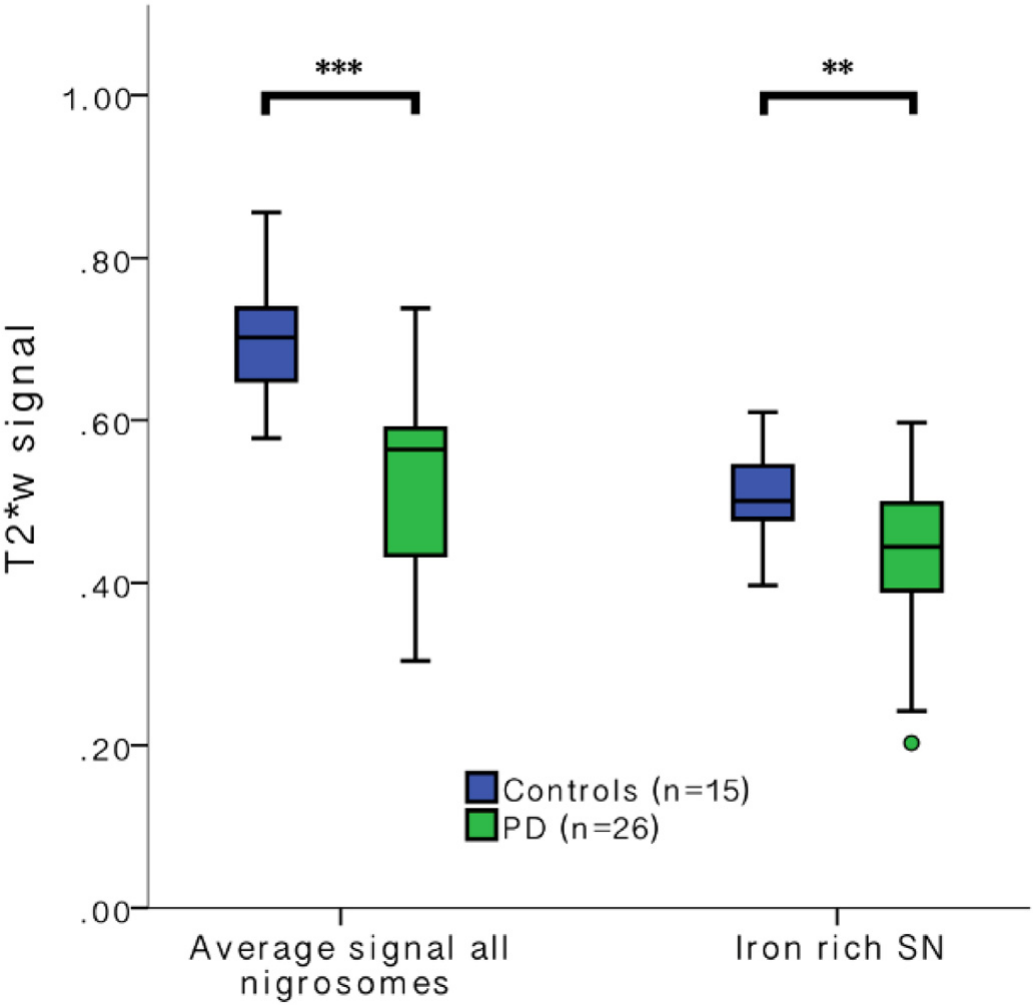
Nigrosomal and iron-rich SN T2*w signal in controls and PD. Box and whiskers plot of T2*w signal (normalised to WM in brainstem tegmentum) in all the nigrosomes and the surrounding iron-rich SN in Controls and PD. (***p < 0.001, **p < 0.01, Mann-Whitney *U* test).

The average T2*w signal in all nigrosomes was higher than in the iron-rich SN in both, controls and PD, reflecting the brighter appearance observed visually. There was a significant PD induced signal reduction for all nigrosomes combined and also in the SN excluding nigrosomes (Fig. 2). The absolute and relative (in relation to controls) signal reduction within all nigrosomes was greater than in the adjacent substantia nigra (relative median signal reduction in all nigrosomes of PD patients: 19.4% [IQR = 24%, min = −4%, max = 59%] vs. median signal reduction in the substantia nigra without nigrosomes of PD patients 11.1% [IQR = 24%, min = −19%, max = 59%], Mann-Whitney *U* test *p* = 0.017). When interrogating the data using a univariate analysis of variance with the T2*w signal of the substantia nigra/nigrosomes as dependent variable and group and NS/SN as independent variable we found a positive interaction of group with NS/SN (F(1,81) = 5.59, *p* = 0.021, partial eta squared = 0.067).

**Fig. 3:**
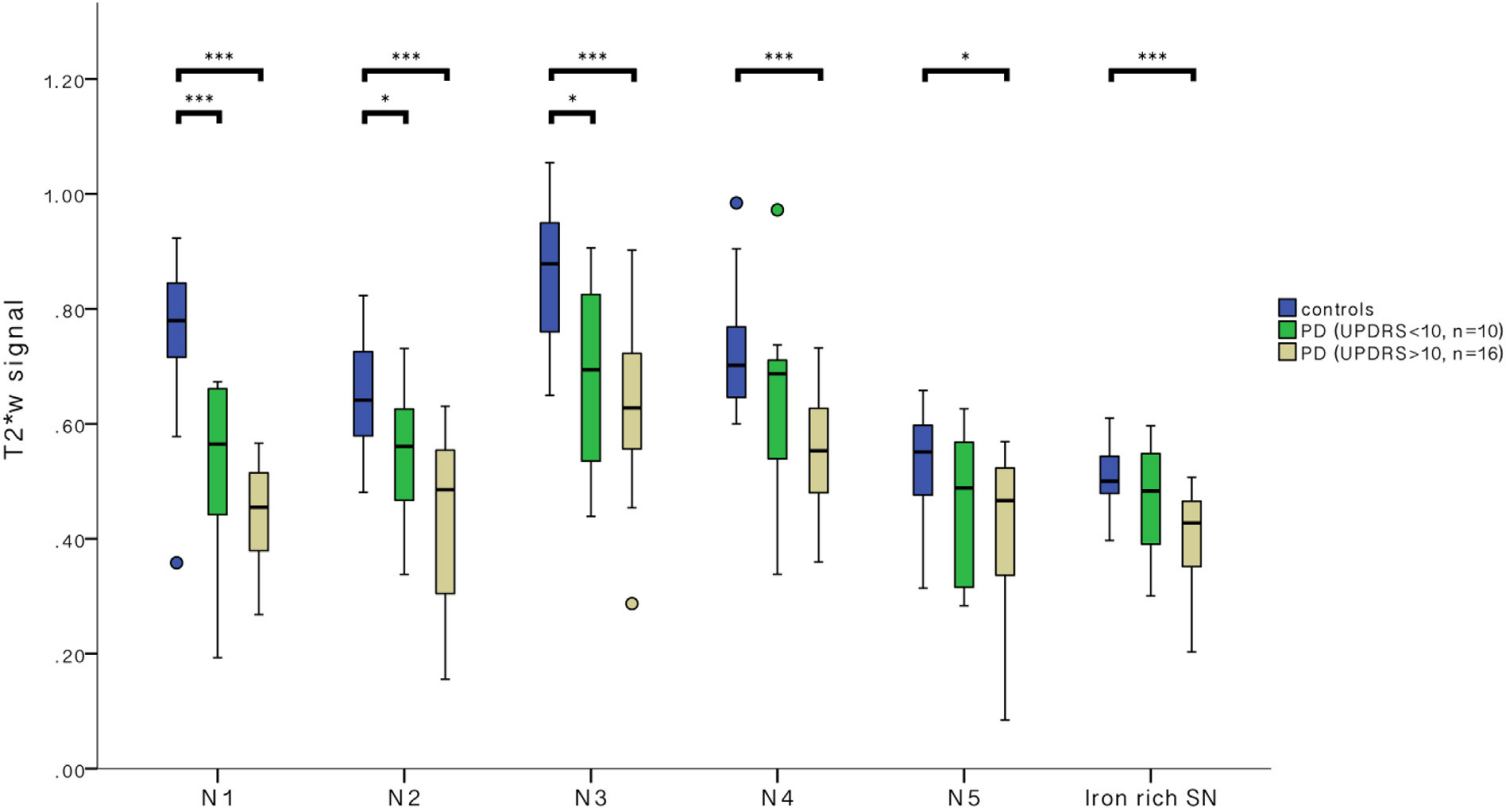
PD severity dependence of T2*w signal of the individual nigrosomes. Variation of T2*w signal (normalised to adjacent WM) in the different nigrosomes and the iron-rich SN in controls (blue) patients with early PD (green, UPDRS ≤ 10) and patients with mild to moderate PD (beige, UPDRS = 11–47). Significant differences between groups using Mann Whitney U test in each region of interest indicated: ***p < 0.001, *p < 0.05. (For interpretation of the references to colour in this figure legend, the reader is referred to the web version of this article.)

The relative signal loss (in relation to controls) in the individual nigrosomes was greatest in N1 (Median signal reduction by 37.2% [IQR = 19%, min = 14%, max = 75%]) and smallest in the iron-rich SN (see above: median signal reduction by 11.1%, *p* < 0.001). There was less relative signal loss in N2–N5 when compared to N1 (significantly highest PD induced signal loss in N1 when comparing vs. N2–N5 all *p* > 0.05, Bonferroni corrected). This is consistent with PD induced preferential darkening of the nigrosomes, especially of N1 (Fig. 3).

### 3.5 Variation in T2*w signal of nigrosomes and iron-rich SN with UPDRS

**Fig. 4:**
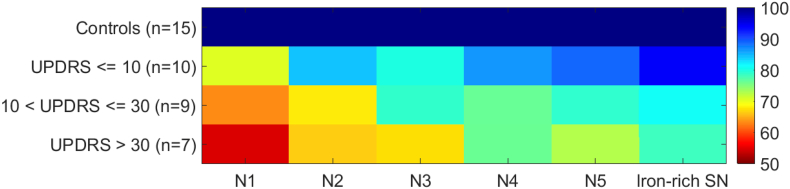
Colour chart representation of PD severity dependence of T2*w signal in the different nigrosomes. The T2*w signal of the individual nigrosomes was normalised to the average signal measure in control subjects and relative signal loss is demonstrated on a colour scale with red indicating the most prominent signal reduction. PD patient's were grouped into three severity groups according to the UPDRS. The largest signal reduction in relation to the signal in controls is seen in N1, whereas the signal change in the other nigrosomes and the iron-rich SN is smaller. The figure is in accordance of the style of the original illustration which was published by Damier et al., 1999a, Damier et al., 1999b when introducing the concept of different nigrosomes with disease severity dependent vulnerability.

When comparing the T2*w signal change in PD patients with UPDRS > 10 to very early PD patients (UPDRS ≤ 10) and the control group, there is increasing signal loss from the early to the later PD stages for all Nigrosomes and the iron-rich SN (Fig. 3). The prominent signal loss in N1 in comparison to the other nigrosomes and the iron-rich SN is also descriptively visualized in a colour chart reflecting the T2*w signal change in the individual nigrosomes and iron-rich SN in relation to controls (Fig. 4).

**Fig. 5:**
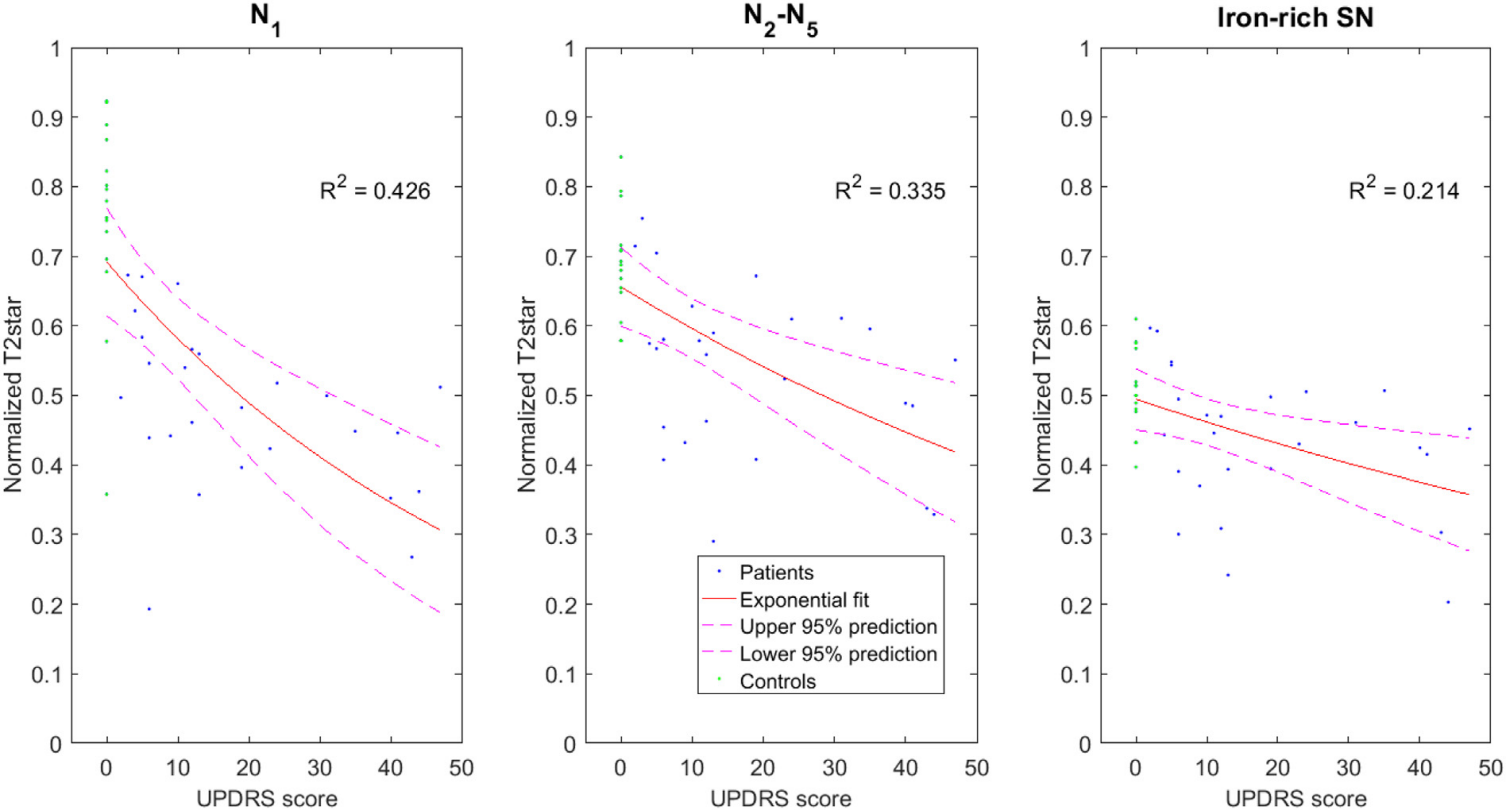
Correlation of nigrosomal T2* signal with UPDRS. T2*w signal in a) N1 b) averaged across N2–N5 and c) in the iron-rich SN plotted against UPDRS score for patients and controls (UPDRS = 0). An exponential fit is superimposed and the R-squared value is given as a measure of the goodness of fit (including controls).

Using simple exponential regression analysis and assuming controls to have UPDRS of 0, the highest correlation between T2*w signal and UPDRS score was seen in N1 (R^2^ = 0.43) with a lower correlation in N2–5 (averaged) and the iron-rich SN (R^2^ = 0.34, R^2^ = 0.21 respectively) (Fig. 5). Using a linear regression analysis instead did show the same trend (R^2^ = 0.40, R^2^ = 0.32, R^2^ = 0.21 for N1, N2–5 and iron-rich SN respectively). To account for the fact that we did not measure the UPDRS in our controls and assumed a UPDRS of 0 we also performed a simple exponential regression analysis including only data from PD patients (*n* = 26). This revealed a significant negative association between T2*w signal in N1 and UPDRS (F_(1,25)_ = 5.46, *p* = 0.026, R^2^ = 0.19), but the correlation did not reach significance for N2–5 or the iron-rich SN. There was no significant correlation of disease duration with the signal in any of the nigrosomes. There was a significant correlation of signal in N3 with the HY score (F_(1,25)_ = 4.89, *p* = 0.037, R^2^ = 0.17) but no significant correlation of HY score with the other nigrosomes. This result did not pass multiple comparisons correction. We did not find a significant correlation of normalised and non-normalised T2*w signal in any of the nigrosomes or the iron-rich SN with age in our control cohort.

## 4 Discussion

Using ultra-high field 7T MRI with high resolution T2*w imaging we were able to identify all five nigrosomes in vivo in healthy controls using a qualitative descriptor on the range of visibility and identification confidence. We found that the nigrosomes were less visible in the PD patients than in controls. N1 was identified with highest confidence, and yielded the highest sensitivity and specificity for PD pathological changes. The T2*w signal in N1 was also inversely correlated with disease severity (UPDRS).

MRI assessment of N1 as a sensitive and specific diagnostic marker of PD is a relatively new concept introduced by our research group four years ago using 7T MRI and 3 T MRI high resolution T2*w MRI. The validity of this imaging test has been confirmed by other research groups using both 3 T (Cosottini et al., 2015; Mahlknecht et al., 2017; Noh et al., 2015; Reiter et al., 2015; Schwarz et al., 2014) and 7T (Blazejewska et al., 2013; Cosottini et al., 2014, Cosottini et al., 2015) iron sensitive sequences. A previous in vivo study confirmed an internal 3-tier complex organisation of the SN using 7T MRI (Cosottini et al., 2014). In the study the importance of the internal architecture of the SNpc and the presence of several high-signal intensity T2*w structures has been demonstrated and related to pathological changes in PD. Although the authors speculated that the signal might relate to the presence of nigrosomes, the individual nigrosomes were not separately demonstrated or assessed. A recent post mortem study comparing histopathological PD induced nigrosome alterations with 9.4 T MRI demonstrated that all nigrosomes can be visualized on high resolution T2w MRI in patients post mortem with and without PD (Massey et al., 2016). However, the ability to visualize all five nigrosomes in vivo has not been demonstrated previously.

The results of our study suggest that ultra-high-resolution iron-sensitive MRI can track the histopathologically known spatiotemporal progression of dopaminergic cell loss from N1 to the other nigrosomes. Damier described a sequential loss of dopaminergic neurons with N1 most prominently affected in the early stage of the disease followed by N2, N4, N3, N5 and then the iron-rich SN in later disease stages (Damier et al., 1999b). Correspondingly we found that N1 showed the highest correlation of T2*w signal loss with UPDRS and the iron-rich SN one of the lowest correlations in our sample. The signal loss in nigrosomes is probably related to iron deposition or other intrinsic signal alterations rather than due to nigrosome volume loss, as a previous study has shown that NS volumes are not significantly changed throughout the disease (Massey et al., 2016). Alterations to the SN iron content may be the result of PD induced loss of neuromelanin pigment. We recently have been able to demonstrate a correlation of UPDRS with neuromelanin related SN signal (Schwarz et al., 2017). Neuromelanin is involved in the intra-cellular iron metabolism and PD induced alteration of neuromelanin content and structure may lead to a pathological increase in intra- or extracellular iron (Zecca et al., 2006; Zucca et al., 1996).

There is a slight difference in degree of signal change we found in the individual nigrosomes and the neuronal loss described by Damier et al. (1999b). This might due to a non-linear relationship of neuronal loss and SN iron content or due to the different ranges of diseases severities of our (disease duration <8 years) and Damier's study (disease duration <32 years).

Multiple publications have confirmed the ability to demonstrate disease severity related changes by estimating SN iron content of the whole SN or the SNpc using iron sensitive MRI sequences or quantitative susceptibility mapping (Fu et al., 2016; Lotfipour et al., 2012; Martin et al., 2008; Wallis et al., 2008). The signal alterations have been shown to correlate with disease severity measures such as motor severity (Bunzeck et al., 2013; Martin-Bastida et al., 2017; Wallis et al., 2008; Zhang et al., 2010). Progressive iron related signal reduction over the course of the disease can be seen in longitudinal studies (Ulla et al., 2013; Wieler et al., 2015). However, there has been considerable variation in ROI definition in the literature and no definite imaging features allow an easy segmentation of the SN into pars compacta and pars reticulata. This is a well-recognised limitation of nigral MRI as a biomarker of PD (Schwarz et al., 2013). In this study we were able to demonstrate that the PD induced iron related T2*w signal loss was most prominent in N1, suggesting that outlining N1 might improve accuracy to track disease severity related changes particularly in the very early stage of the disease.

### 4.1 Limitations

Segmenting N1-N5 can be challenging especially in PD as the physiological bright nigrosomal T2*w signal is lost. However, we achieved fair to good inter- and intra-rater reliability confirming the validity of this method. Semi-quantitative measurement of the T2*w signal of each nigrosome was performed comparing signal from each nuclei to adjacent WM. Improved estimation of the SN iron content may be achieved by quantitative assessment of signal using multi-echo T2*w techniques or quantitative susceptibility mapping. Quantitative estimation of the susceptibility change of the nigrosomes during disease progression would be very valuable in the future, but technical challenges such as patient motion need to be overcome to be able to acquire high resolution quantitative susceptibility data of these small features within the SN in vivo.

We assessed the patients' UPDRS whilst they were fully medicated which limits the accuracy to capture individual motor and non-motor symptoms of PD and also limits the reliability of UPDRS as measure of disease severity. A further limitation is that the UPDRS in controls was assumed to be 0 without assessment. This is presumably an underestimation of the true UPDRS in controls (Wilson et al., 2002). However, the demonstrated UPDRS values of our PD patient cohort “on medication” are also expectantly lower than the UPDRS “off treatment”.

## 5 Conclusion

All Nigrosomes 1–5 can be detected using high resolution 7T T2*w MRI and are all less confidently identified in PD patients when compared to controls. The PD induced T2*w signal reduction was greater in the nigrosomes than in the iron-rich SN with the significantly highest signal reduction in N1. The graded T2*w signal alterations in the nigrosomes match previously reported differential pathophysiological disease effects of PD on nigrosomes and the iron-rich SN at different stages of the disease. Comparing all nigrosomes and the iron-rich SN, MRI signal interrogation in N1 has the most favourable biomarker characteristics as it exhibited the largest PD induced signal change even in the earliest stages of the disease. N1 was identified with the highest confidence and correlated best to UPDRS as a measure of disease severity.

## Appendix A Supplementary data

Supplementary material Image 1
