# Supplementary material for: Parkinson's disease related signal change in the nigrosomes 1–5 and the substantia nigra using T2* weighted 7T MRI
Source: Neuroimage Clin. 2018 May 24;19:683–9. doi: 10.1016/j.nicl.2018.05.027 (PMC5986169; doi:10.1016/j.nicl.2018.05.027)
Supplement: Supplementary file 1 — Supplementary material [file mmc1.docx]

Supplement:

Table of participants with individual measures:

| Part. No. | ACE | MMSE | PD duration [years] | UPDRS | HY score | PD_HV (1=PD) | SEX (1=f) | Age [years] |
| --- | --- | --- | --- | --- | --- | --- | --- | --- |
| 1 | 83 | 28 | 5 | 43 | 3 | 1 | 0 | 86.92 |
| 2 | 97 | 30 | 7 | 40 | 1 | 1 | 1 | 66.81 |
| 3 | 99 | 29 |  |  |  | 0 | 1 | 61.26 |
| 4 | 83 | 28 | 0.5 | 23 | 1 | 1 | 0 | 62.83 |
| 5 | 98 | 29 | 1.5 | 11 | 1 | 1 | 0 | 65.77 |
| 6 | 97 | 29 |  |  |  | 0 | 1 | 68.07 |
| 7 | 99 | 30 |  |  |  | 0 | 1 | 64.83 |
| 8 | 100 | 30 |  |  |  | 0 | 0 | 59.85 |
| 9 | 100 | 30 |  |  |  | 0 | 1 | 64.8 |
| 10 | 98 | 30 | 0.3 | 35 | 2 | 1 | 0 | 64.45 |
| 11 | 98 | 30 | 5 | 31 | 1 | 1 | 0 | 62.99 |
| 12 | 92 | 29 | 4 | 24 | 2 | 1 | 0 | 70.61 |
| 13 | 100 | 30 |  |  |  | 0 | 1 | 67.45 |
| 14 | 93 | 29 | 1 | 12 | 1 | 1 | 1 | 67.28 |
| 15 | 95 | 26 |  |  |  | 0 | 0 | 59.72 |
| 16 | 100 | 30 | 3.5 | 6 | 1 | 1 | 1 | 62.41 |
| 17 | 99 | 30 |  |  |  | 0 | 1 | 62.84 |
| 18 | 82 | 27 | 2 | 19 | 1 | 1 | 0 | 55.89 |
| 19 | 72 | 24 | 0.1 | 41 | 2 | 1 | 0 | 76.38 |
| 20 | 89 | 25 | 1.2 | 12 | 1.5 | 1 | 0 | 58.3 |
| 21 | 90 | 28 | 1.5 | 3 | 1 | 1 | 0 | 63.09 |
| 22 | 91 | 28 | 0.1 | 2 | 1.5 | 1 | 1 | 61.35 |
| 23 | 88 | 24 |  |  |  | 0 | 1 | 67.41 |
| 24 | 88 | 21 | 1.1 | 13 | 1.5 | 1 | 0 | 71.34 |
| 25 | 88 | 30 | 1.5 | 4 | 1 | 1 | 0 | 66.16 |
| 26 | 94 | 30 |  |  |  | 0 | 0 | 64.96 |
| 27 | 92 | 28 | 6 | 9 | 1 | 1 | 0 | 51.91 |
| 28 | 83 | 26 | 3 | 5 | 1.5 | 1 | 1 | 76.64 |
| 29 | N/A | N/A | 4 | 13 | 1.5 | 1 | 1 | 67.18 |
| 30 | 92 | 25 | 1 | 6 | 1 | 1 | 0 | 64.34 |
| 31 | 94 | 25 | 3 | 5 | 1.5 | 1 | 0 | 66.33 |
| 32 | 93 | 28 | 8 | 6 | 1.5 | 1 | 1 | 40.09 |
| 33 | 91 | 29 |  |  |  | 0 | 1 | 60.45 |
| 34 | 98 | 30 |  |  |  | 0 | 1 | 79.52 |
| 35 | 93 | 30 | 2 | 44 | 2 | 1 | 0 | 68.83 |
| 36 | 98 | 29 | 1 | 19 | 1 | 1 | 1 | 62.04 |
| 37 | 93 | 28 | 1.5 | 47 | 2 | 1 | 0 | 68.68 |
| 38 | 98 | 29 | 1 | 10 | 1 | 1 | 0 | 56.38 |
| 39 | 92 | 29 |  |  |  | 0 | 0 | 67.07 |
| 40 | 97 | 29 |  |  |  | 0 | 0 | 69.82 |
| 41 | 99 | 30 |  |  |  | 0 | 1 | 61.35 |
